# Neuronal-surface antibodies are uncommon in patients with a final diagnosis of sporadic Creutzfeldt-Jakob disease: a systematic review and meta-analysis

**DOI:** 10.3389/fimmu.2026.1966990

**Published:** 2026-10-06

**Authors:** Najeeb Qadi, Amaal Aldakheel, Eslam Shosha

**Affiliations:** 1 Neuroscience Centre of Excellence, King Faisal Specialist Hospital and Research Centre, Riyadh, Saudi Arabia; 2 College of Medicine, Alfaisal University, Riyadh, Saudi Arabia; 3 Division of Neurology, Department of Medicine, McMaster University, Hamilton, ON, Canada

**Keywords:** autoimmune encephalitis, Creutzfeldt-Jakob disease, meta-analysis, neuroimmunology, neuronal antibodies, prion disease, rapidly progressive dementia

## Abstract

**Background:**

Neuronal antibodies detected during evaluation of rapidly progressive dementia may indicate treatable autoimmune encephalitis or incidental seroreactivity in prion disease. We estimated their frequency in sporadic Creutzfeldt-Jakob disease (sCJD), separately from diagnostic yield in suspected CJD.

**Methods:**

Under a registered protocol (PROSPERO CRD420261458659), MEDLINE/PubMed, Embase and CENTRAL were searched on 12 July 2026 and with expanded antibody terms on 13 September 2026. Screening, extraction and JBI appraisal were performed independently in duplicate with third-reviewer adjudication. The primary outcome was neuronal-surface/synaptic antibody positivity in datasets with standardized testing. We report the aggregate proportion with an exact 95% confidence interval and model-based summaries from logistic-normal GLMM and beta-binomial models, which estimate different quantities.

**Results:**

Forty-three reports were retained, including six independent datasets with 241 tested patients. All four low-level positives arose in one cohort: aggregate 4/241 (1.66%; 95% CI 0.45–4.19), GLMM 0.36% (lower bound <0.01%, upper bound 3.38%), and beta-binomial 1.21% (0.07–19.67). Restricting positivity to an identified neuronal-surface antigen gave 3/241 (1.24%; 0.26–3.59). Excluding two datasets at high risk of bias gave 4/154 (2.60%; 0.71–6.52); excluding the dataset with inseparable diagnostic-certainty groups gave 4/220 (1.82%; 0.50–4.59). No antibody was detected in 51 pathologically confirmed patients with directly linkable testing results from two non-overlapping datasets (0/51; exact upper 95% bound 7.0%). This selected subset does not encompass all pathologically confirmed patients or exclude antibody positivity in confirmed disease.

**Conclusions:**

Neuronal-surface antibodies were uncommon on the assays used, but estimates depend on antibody definition, denominator composition and model assumptions. Frequency in sCJD does not establish the probability of autoimmune disease after a positive antibody result. Interpretation requires the antibody identity, specimen, assay confirmation, phenotype and strength of prion evidence; autoimmune encephalitis and sCJD can coexist.

**Systematic review registration:**

https://www.crd.york.ac.uk/PROSPERO/view/CRD420261458659, identifier CRD420261458659.

## 1 Introduction

Sporadic Creutzfeldt-Jakob disease (sCJD) is a rapidly progressive and fatal prion disease. Antemortem diagnosis relies on the clinical phenotype, characteristic diffusion-weighted magnetic resonance imaging, electroencephalography and cerebrospinal-fluid (CSF) biomarkers, particularly real-time quaking-induced conversion (RT-QuIC), while neuropathology remains the reference standard for definite disease (1). The principal clinical urgency is not that CJD itself is treatable, but that several disorders capable of mimicking it are.

Autoimmune encephalitis is the most therapeutically important mimic. Patients may present with rapidly progressive cognitive decline, psychiatric symptoms, seizures, movement disorders or myoclonus, and conventional MRI or EEG can overlap with CJD. Unlike prion disease, many autoimmune encephalitides respond to timely immunotherapy (2). A neuronal-antibody result obtained in this setting is therefore rarely a binary choice between treating autoimmune encephalitis and dismissing incidental seropositivity. In practice it prompts confirmatory testing and a simultaneous reassessment of both the autoimmune and the prion evidence, and the clinical meaning of the result depends on the population in which the test is interpreted.

Two distinct questions arise. First, among patients who ultimately have genuine sCJD, how often are neuronal antibodies detectable? This addresses whether antibody positivity represents incidental seroreactivity, a secondary immune response to neuronal injury, or true dual pathology. Second, among patients initially investigated for suspected CJD, how often does antibody testing uncover a treatable alternative diagnosis? This is a diagnostic-yield question. Combining these populations in a single denominator obscures both.

A recent review by Bilavu and colleagues summarized antibody-positive CJD cases and characterized their clinical features, including several non-neuronal antibodies, but did not provide a denominator-based estimate because antibody-negative tested cohorts were not synthesized (3). The present review addresses the complementary denominator-based question and explicitly separates patients with a final diagnosis of sCJD from patients in the suspected-CJD diagnostic work-up.

We therefore aimed primarily to estimate the frequency of prespecified neuronal antibodies among patients with a final clinical or pathological diagnosis of sporadic CJD. Secondary aims were to characterize antibody type, sample and assay features; to examine sensitivity to antibody specificity, study design and study quality; and to synthesize the diagnostic significance of neuronal-antibody testing in patients initially suspected of CJD.

## 2 Methods

### 2.1 Protocol, registration and reporting

The review protocol was prepared in advance and registered in PROSPERO (CRD420261458659). Reporting follows the PRISMA 2020 statement (4). The primary estimand was clarified before final quantitative synthesis to distinguish antibody frequency in patients with a final diagnosis of sCJD from diagnostic yield in suspected CJD; this clarification did not change the underlying eligibility rule requiring an extractable sCJD antibody numerator and denominator for frequency estimation. Three matters arose during peer review and are disclosed here. First, the stated restriction of the frequency synthesis to probable or definite disease did not fully describe the Gurram dataset, whose antibody-tested subset also contained inseparable possible or unclassifiable diagnoses; this exception is stated in Section 2.2 and examined by a *post hoc* exclusion analysis, and the broader inclusion is not described as prespecified. Second, a *post hoc* clarification required qualitative reports to name at least one eligible neuronal target with its result. Applying this criterion to both searches removed three originally retained reports; a fourth was removed under the full-report criterion because it was a conference abstract (Section 3.1). Third, three sensitivity analyses (exclusion of datasets at high risk of bias, exclusion of the Gurram dataset, and an alternative model specification) were added *post hoc* and are identified as such in Section 2.7.

### 2.2 Eligibility criteria and antibody definition

Eligible primary reports included prospective or retrospective cohorts, cross-sectional or case-control studies, case series and single case reports describing neuronal-antibody testing in patients with possible, probable or definite sporadic CJD according to recognized criteria. Genetic/familial, iatrogenic and variant CJD were excluded unless a sporadic subgroup was separately extractable. For the frequency synthesis, the final diagnosis had to remain sCJD, whether established clinically (probable or definite by recognized criteria, including RT-QuIC where used) or neuropathologically; the term “final clinical or pathological diagnosis of sCJD” is used throughout to make explicit that several contributing cohorts were probable or clinically diagnosed rather than neuropathologically definite. Gurram et al. also included possible and unclassifiable clinical diagnoses, and the diagnostic-certainty distribution of the 21 antibody-tested patients could not be separated; this exception limits the certainty of the frequency denominator and is examined by excluding that cohort in a *post hoc* sensitivity analysis (Section 3.4). Patients ultimately found to have autoimmune encephalitis or another mimic were analyzed only under the secondary diagnostic-significance objective.

Single case reports were eligible for qualitative synthesis only when they contained at least one specifically named eligible neuronal target with its result, sufficient information to establish sporadic CJD, and an extractable patient-level result; an unspecified “autoimmune” or “paraneoplastic” panel without named targets was not sufficient, and conference abstracts without a full report were not eligible. This operational rule was applied consistently to the original and supplementary searches. Case reports were not treated as frequency studies.

The prespecified antibody definition included neuronal-surface/synaptic antibodies (including NMDAR, LGI1, CASPR2, GABA-A and GABA-B receptors, AMPAR, DPPX, IgLON5, glycine receptor and metabotropic glutamate receptors) and recognized intracellular/onconeural antibodies (including Hu/ANNA-1, Yo, Ri, CV2/CRMP5, Ma2, amphiphysin and GAD65). Because the included studies span two decades of assay practice, the primary outcome was defined operationally as positivity on the neuronal-surface/synaptic antibody assays used in the source studies, including legacy VGKC-complex assays; a prespecified specificity-focused sensitivity analysis restricted positivity to antibodies with an identified neuronal-surface antigen. VGKC-complex positivity without LGI1 or CASPR2 specificity is not equivalent to an identified neuronal-surface antigen and is regarded as clinically irrelevant, which is the reason the primary analysis is accompanied by a prespecified analysis excluding it. AQP4, MOG, thyroid antibodies, ganglioside antibodies, anti-neurofilament antibodies, oligoclonal bands, IgG index, cytokines, PRNP testing and non-antibody CJD biomarkers were not counted as neuronal-antibody outcomes. AQP4 is a surface antigen but is astrocytic rather than neuronal and was therefore excluded.

Review question (PICOS). Population: patients with a final clinical or pathological diagnosis of sporadic Creutzfeldt-Jakob disease, and separately, patients under investigation for suspected CJD. Intervention (exposure): testing for neuronal antibodies in serum or cerebrospinal fluid. Comparator: none for the primary frequency outcome; the two populations are reported separately and are not pooled. Outcomes: primary, the proportion of tested patients positive on neuronal-surface/synaptic antibody assays; secondary, antibody target, specimen and assay characteristics, and the diagnostic significance of antibody testing in suspected CJD. Study designs: prospective or retrospective cohorts, cross-sectional and case-control studies, and case series with an extractable numerator and denominator; single case reports contributed to qualitative synthesis only. Reporting followed PRISMA 2020.

### 2.3 Information sources and search

MEDLINE (via PubMed), Embase and the Cochrane Central Register of Controlled Trials (CENTRAL) were searched on 12 July 2026. No date, language or study-design filters were applied. Search concepts combined CJD/prion disease/rapidly progressive dementia with neuronal antibodies/autoimmune encephalitis. The antibody block of the original search comprised generic neuronal-antibody and autoimmune-encephalitis terms together with NMDAR, LGI1 and CASPR2, and was therefore narrower than the eligibility definition in Section 2.2. A supplementary search was therefore executed on 13 September 2026 in PubMed (328 records), Embase.com (901 article records and 4 clinical-trial records) and CENTRAL (4 records), without date, language or study-design limits, with the antibody block expanded to cover every eligible named antigen and its synonyms (autoantibod*, NMDA receptor, glycine receptor/GlyR, GAD/GAD65, IgLON5, AMPAR, GABA-A/GABA-B receptor, DPPX/DPP6, mGluR, Hu/ANNA-1, CRMP5/CV2, Ma2, amphiphysin, Yo/PCA-1 and Ri/ANNA-2). The supplementary search retrieved 1,237 records; publication-level deduplication removed 248, leaving 989 unique records, of which 652 matched publications already in the original screening set and 337 were additional records that entered screening (Section 2.4). Backward and forward citation searching and conference-proceeding review supplemented the database search. The complete search string for each database for the original search (**Supplementary Table 1**), the reconciled table of full-text decisions (**Supplementary Table 2**), the risk-of-bias appraisal (**Supplementary Table 3**) and the statistical synthesis details are provided in **Supplementary Material 1**; the executed supplementary-search strategies, raw exports and record-level reconciliation are provided as **Supplementary Data 2**.

### 2.4 Study selection and reviewer agreement

After de-duplication, two reviewers (NQ and ES) independently screened titles/abstracts and full texts. Uncertain records proceeded to full text rather than being excluded on incomplete abstracts. Disagreements were resolved by discussion and, when required, by a third reviewer (AA). For the original search, title/abstract agreement was 87.7% (Cohen’s κ = 0.717, 95% CI 0.660–0.773), with 85 disagreements resolved by the third reviewer; agreement at full-text screening was substantial (Cohen’s κ = 0.79, 95% CI 0.68–0.89), with 15 residual disagreements resolved by the third reviewer. The 337 additional records from the supplementary search were screened by the same two reviewers using the identical independent, duplicate procedure and third-reviewer adjudication; agreement was not separately quantified for this subset.

### 2.5 Data extraction and overlap adjudication

Data were extracted independently in duplicate using a piloted spreadsheet. Variables included study design, CJD diagnostic certainty and its basis (neuropathology, RT-QuIC, clinical criteria), specimen (serum, CSF or both), assay method, antibody targets, number of sCJD patients tested, number antibody-positive, antibody titer/confirmation where available, final alternative diagnoses and immunotherapy response. Raw source counts were kept separate from primary-synthesis counts. Earlier case reports or conference abstracts contained within later cohorts were retained descriptively but were not double-counted.

Complex denominators were adjudicated at the paper level before final quantitative synthesis and are set out dataset by dataset in **Supplementary Table 5**. For Rossi et al., although 150 patients had undergone clinically directed antibody testing, that testing was non-uniform across antibody targets: VGKC-complex was requested in 119 patients, NMDAR in 77 and glycine receptor in 6. Only the 82 retrieved sera underwent systematic testing with the standardized panel (NMDAR, glycine receptor, VGKC-complex, LGI1 and CASPR2). The primary synthesis therefore used the 82-patient subset, which is the only denominator in that report corresponding to standardized ascertainment of the review outcome. Of these 82 patients, 42 were postmortem-confirmed and 40 were clinically diagnosed; the report does not allow the four antibody-positive patients to be cross-tabulated with pathological confirmation, so the diagnostic certainty of the positive patients is unknown and the 42 postmortem-confirmed patients cannot be assigned an antibody status individually. Results from the clinically directed 150-patient experience were retained descriptively and were not treated as an equivalent frequency denominator (5). Across the combined clinical and retrospective testing experience, seven antibody-positive patients were described in that report: four on clinically directed testing and four on systematic retesting, one of whom had been identified previously. This adjudication applies the eligibility rule stated in Section 2.2, which requires an extractable numerator and denominator for the antibodies actually tested; it was made before the final synthesis and was not prompted by the observed result. For Gurram et al., the 21 patients tested with the autoimmune-encephalitis panel contributed the primary denominator, while the overlapping 19-patient paraneoplastic panel was retained separately (6). For Lazar et al., neural-specific autoantibody panels were reported separately by specimen: serum in 71 patients and cerebrospinal fluid in 79, with no neural-specific autoantibody detected in either compartment among the CJD cases (nine patients had serum reactivities against targets outside the primary neuronal-surface encephalitis-antibody definition, which contribute only to the exploratory analysis); the number of unique patients tested in either compartment was not reported. Because the primary outcome permits serum or cerebrospinal fluid testing, the larger fully documented single-compartment denominator (79) was used; this avoids summing overlapping specimen groups and gives a lower aggregate proportion than the serum denominator would. Substituting the serum denominator (71) changes the aggregate proportion from 1.66% to 1.72% and does not alter any conclusion. Ong et al. contributed two autopsy-confirmed patients with an explicit antibody denominator and result, which distinguishes the report from a single case report; a prespecified sensitivity analysis restricted to datasets with at least 10 tested patients examines the influence of the two smallest datasets (Section 3.4). Two further multi-patient reports required specific denominator adjudication. Mackay et al. reviewed 100 pathologically confirmed sporadic CJD cases seen by the UK National CJD Research and Surveillance Unit, of whom six had NMDAR antibody results available; two were positive at low titer in serum (1:80) and cerebrospinal fluid was negative in all six. Patients in the United Kingdom with suspected CJD are commonly referred to one of these two national centres, and both series were tested at the same reference laboratory. The two reports therefore draw on the same national referral stream and patient overlap cannot be excluded, so Mackay et al. was retained descriptively rather than included in the quantitative synthesis; its relevance to the pathologically confirmed subgroup is addressed in Section 3.3. Chen et al. contributed 8 patients: of 68 patients with sporadic CJD, eight non-biopsied cases underwent testing for neuronal surface antigens (LGI1, CASPR2, NMDA and glycine receptors) together with intracellular antigens, and all were negative. The eight autoimmune-encephalitis patients described in the same report are mimics of sporadic CJD and do not contribute to the sCJD denominator.

### 2.6 Risk of bias and certainty

Risk of bias was assessed using design-matched JBI tools: the Checklist for Case Series (10 items) for Gurram 2023, Grau-Rivera 2014, Rossi 2015, Chen 2016 and Ong 2015, and the Checklist for Case Control Studies (10 items) for Lazar 2022. These datasets were not designed as prevalence studies. Because our synthesis addresses frequency, we also examined sampling frame, systematic ascertainment, completeness and consistency of antibody testing, and clarity of the CJD reference standard. These considerations complement the design-matched ratings; no separate JBI prevalence-tool score was calculated. Incomplete testing and inseparable diagnostic-certainty groups, particularly the 21 of 25 patients tested in Gurram et al., are described in **Table 1** and Section 3.6. Case reports contributed clinical context rather than frequency estimates. Small multi-patient datasets with explicit testing denominators could enter the quantitative synthesis, but remained susceptible to selection and reporting bias. Sensitivity analyses examined exclusion of high-risk datasets and datasets with fewer than 10 tested patients (Section 2.7). Checklist counts were not converted into an unvalidated numerical quality score. Dataset-level ratings and their rationale are provided in **Supplementary Table 3**.

**Table 1:** Independent multi-patient datasets contributing to the primary frequency analysis.

Study	sCJD population and basis of diagnosis	Specimen	n tested	n positive	Neuronal-surface assay scope	Key denominator note
Gurram 2023 (6)	Clinical sCJD cohort: parent cohort of 25 after follow-up: 1 definite, 20 probable, 2 possible, 2 unclassifiable; 2017 CDC criteria; RT-QuIC not performed; diagnostic certainty of the 21 tested patients not separable	serum	21	0	AE panel (NMDAR, CASPR2, LGI1, GABA-B, AMPA); all negative	21/25 underwent the AE panel; possible/unclassifiable diagnoses cannot be separated within the tested subset (Sections 2.2, 3.4); coverage limitation from a frequency standpoint (Section 3.6)
Grau-Rivera 2014 (41)	Definite (neuropathologically confirmed) CJD from a reference-centre series; sporadic subtype not separately stated; retained as a surveillance-cohort proxy	CSF only	49	0	Comprehensive CSF neuronal-surface panel (LGI1, CASPR2, NMDAR, GlyR cell-based assays plus brain immunohistochemistry)	All 49 neuropathologically confirmed; antibody status directly linkable to pathological confirmation
Lazar 2022 (39)	Definite/probable CJD (case-control); parent cohort of 93: 33 pathologically proven, 60 probable with positive RT-QuIC; certainty split of the tested subsets not separately extracted	Serum (71) and CSF (79)	79	0	Neuronal-surface: 0/71 serum, 0/79 CSF. Exploratory seroreactivity: 9 (5 very low-level GAD65, 2 VGCC, 1 AChR, 1 striated muscle)	Negative for the primary encephalitis-antibody outcome; CSF denominator (79) used, see Section 2.5; nine serum reactivities outside the primary definition contribute only to the exploratory analysis
Rossi 2015 (5)	82 systematically retested: 42 postmortem-confirmed, 40 clinically diagnosed (probable)	Serum (retrieved sera)	82	4	CASPR2 (3, two with GlyR, one with NMDAR); VGKC-complex without LGI1/CASPR2 specificity (1); all low-level	Standardized panel in 82 retrieved sera; positives cannot be cross-tabulated with pathological confirmation; the clinically directed 150-patient experience not used as a denominator (Section 2.5)
Chen 2016 (28)	Clinically diagnosed sCJD 2009 criteria; RT-QuIC not reported; 8 non-biopsied cases	serum and CSF	8	0	Neuronal surface antigens (LGI1, CASPR2, NMDAR, GlyR) plus intracellular antigens; all negative	8 of 68 sCJD patients underwent antibody testing; the 8 autoimmune-encephalitis patients in the same report are mimics and do not contribute
Ong 2015 (40)	Two autopsy-confirmed sCJD (VV2); neuropathology; RT-QuIC positive in 1, not performed in 1	serum	2	0	Legacy VGKC-complex; both negative; additional LGI1 testing negative in patient 2	Antibody-negative; prion-confirmed; antibody status directly linkable to pathological confirmation; assay narrower than modern panels

Denominators are the numbers systematically tested for the prespecified neuronal-surface antibodies. AE, autoimmune encephalitis; AChR, acetylcholine receptor; CJD, Creutzfeldt-Jakob disease; CSF, cerebrospinal fluid; RT-QuIC, real-time quaking-induced conversion; sCJD, sporadic CJD; VGCC, voltage-gated calcium channel; VGKC, voltage-gated potassium channel.

Certainty in the body of evidence was judged across the GRADE domains of risk of bias, imprecision, inconsistency, indirectness and publication bias, applied to a single-proportion outcome; a formal GRADE evidence profile was not constructed, since GRADE is designed for comparative effect estimates. The certainty of the primary frequency estimate was rated low because only six independent multi-patient datasets were available, antibody panels and ascertainment varied, and two of the largest datasets had incomplete or clinically selected antibody testing. Formal funnel-plot asymmetry testing was not performed: the synthesis contained fewer than 10 independent datasets, and case reports are not an appropriate sampling frame for such tests. This low rating reflects, in addition to imprecision, the finding that five of six contributing datasets were at moderate or high risk of bias on formal JBI appraisal.

### 2.7 Statistical analysis

The primary quantitative outcome was the proportion of systematically tested patients with a final clinical or pathological diagnosis of sporadic CJD who were positive on the neuronal-surface/synaptic antibody assays used in the source studies. Given four events across six datasets, five of which were zero-event, the primary summary reported is the aggregate (crude) proportion, that is, the total number of positive patients divided by the total number tested, with an exact Clopper-Pearson binomial interval. This is a descriptive quantity: it treats the 241 patients as a single binomial sample and therefore does not account for between-study differences in source population, selection, specimen, antibody panel or assay method. It is not presented as a meta-analytic prevalence estimate. A random-intercept logistic-normal generalized linear mixed model (GLMM), which retains study-level binomial structure and accounts for within-study uncertainty, was fitted alongside it and is reported with a profile-likelihood interval. Because random-effects models for proportions are weakly identified at this event count, and because the choice of model materially changes the point estimate, a beta-binomial model was also fitted *post hoc* as a check on sensitivity to model specification, and the GLMM with the between-study variance fixed at zero (a common-effect logistic model, which reduces to the aggregate proportion) is noted for comparison. The GLMM summarizes the inverse-logit of the mean study log-odds, whereas the beta-binomial model summarizes the mean of a beta distribution of study probabilities; these are not identical estimands, so differences among the summaries reflect the quantity summarized and the distributional assumptions as well as sparse-data instability. The spread among them is therefore not itself a confidence interval or a formal measure of total uncertainty; it is reported to show that no single figure should be regarded as a precise estimate. Estimation and profile-likelihood details are given in **Supplementary Material 1**. No continuity correction was applied in any model or in the aggregate proportion, and zero-event datasets entered the likelihood directly; the Freeman-Tukey double-arcsine transformation was not used, given documented instability of its back-transformation for proportions near zero with unequal sample sizes (7). Individual-study intervals are exact Clopper-Pearson intervals. Cochran Q and I-squared were computed for description only and were not used for inference; with five zero-event datasets, between-study heterogeneity is imprecisely estimated and an observed value of zero should not be read as evidence of homogeneity (**Supplementary Material 1**). Analyses were performed in Python 3.12 (NumPy 2.3, SciPy 1.17). The complete analysis script, including the validation cases used to verify the implementation, is provided as **Supplementary Data 1**. The analysis was independently re-run from the study-level counts by a second author (AA), who confirmed every quantity reported here.

Two sensitivity analyses were prespecified: a specificity-focused analysis restricting positivity to antibodies with an identified neuronal-surface antigen, and restriction to datasets with at least 10 tested patients. Use of the standardized-testing subset of Rossi et al. was a denominator-adjudication decision applied in the main analysis (Section 2.5), not a separate sensitivity result. Three further analyses were added *post hoc* in response to peer review: exclusion of the datasets rated at high risk of bias (Lazar 2022 and Chen 2016), with a complementary restriction to the single dataset rated at low risk of bias; exclusion of the Gurram dataset, whose tested subset could not be separated by diagnostic certainty; and the beta-binomial model described above. An exploratory analysis counted any reported neural or neuromuscular seroreactivity regardless of antigen target. Single case reports were synthesized narratively. Suspected-CJD diagnostic-yield data were not pooled because referral criteria, diagnostic thresholds and antibody testing strategies were not sufficiently comparable.

### 2.8 Use of a large language model

Large language models (Claude, Anthropic; ChatGPT, OpenAI) assisted with constructing screening workbooks, search and deduplication scripts, analysis-code review, reference formatting, drafting and language editing. During the supplementary search, model assistance also included initial eligibility recommendations and source-data assessment. These preliminary assessments were followed by independent human screening of every additional record, as described in Section 2.4; final eligibility, extraction and risk-of-bias decisions were made by the reviewers. Model-generated recommendations were not treated as independent reviewer decisions or used to calculate reviewer agreement. The authors checked model-assisted text and source interpretations, and AA independently re-ran the statistical analysis as described in Section 2.7. The authors take responsibility for the final manuscript.

## 3 Results

### 3.1 Study selection

The original searches identified 890 records. After removal of 198 duplicates, 692 records underwent title/abstract screening; 463 were excluded and 229 reports were sought for retrieval. One full report was not retrieved, leaving 228 reports assessed for eligibility. Reconciliation of the original log moved that report from an exclusion category to not retrieved and removed four originally retained reports: Suo under the full-report criterion, and Ng, Čiauškaitė and Pimentel under the revised named-target criterion. The original search therefore contributed 36 retained reports and 192 full-text exclusions. The reconciled exclusion categories and report-level corrections are provided in **Supplementary Table 2**. Among 337 additional records from the supplementary search, 313 were excluded at title/abstract screening and 24 reports were retrieved and assessed at full text; 17 were excluded and seven were retained for qualitative synthesis. No additional frequency dataset was identified. Overall, 43 reports were retained: six independent multi-patient datasets contributing to the primary frequency analysis and 37 qualitative-only reports (**Figure 1**).

**Figure 1:**
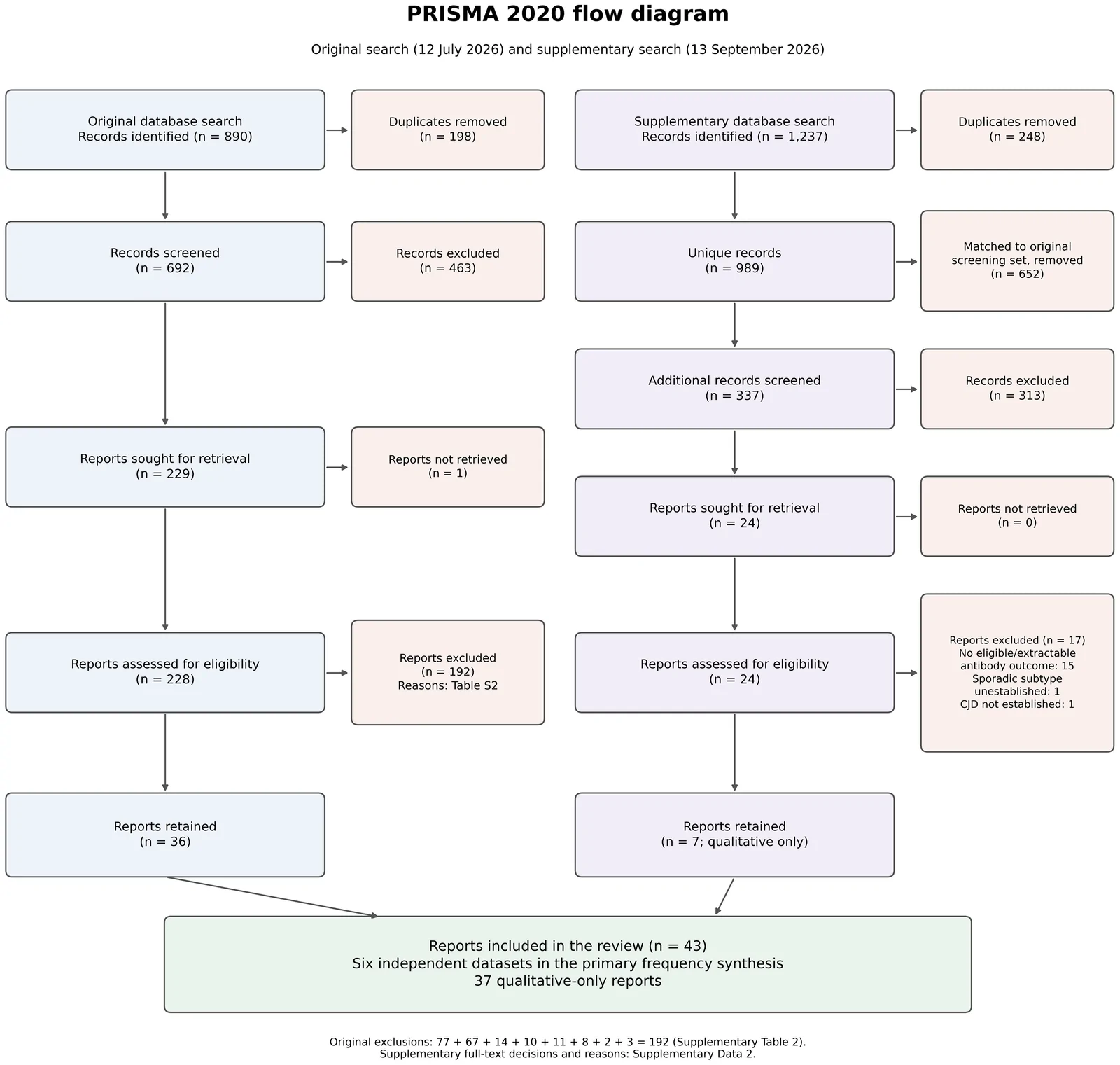
PRISMA 2020 flow diagram for the original and supplementary searches. Original search: 890 records, 692 screened after deduplication, 229 sought for retrieval, 228 assessed (one not retrieved), 36 retained after reconciliation of four originally retained reports. Supplementary search: 1,237 records, 989 unique after deduplication, 652 matching the original screening set, 337 additional records screened, 313 title/abstract exclusions, 24 full-text assessments, 17 full-text exclusions and seven retained. In total 43 reports were retained; six independent multi-patient datasets with a final clinical or pathological diagnosis of sCJD contributed to the primary frequency analysis. Original exclusion reasons and their assigned codes are listed in **Supplementary Table 2**.

### 3.2 Study characteristics and evidence structure

Of the 43 retained reports, six were independent multi-patient datasets eligible for quantitative synthesis and 37 contributed qualitatively only. Four reports cited in the Discussion sit outside the retained set and are used as external context rather than as review evidence: Maat et al., an autopsy-surveillance cohort; van Steenhoven et al., a prospective rapidly progressive dementia (RPD) cohort; Flanagan et al., a series of selected immunotherapy non-responders in which two patients with a final diagnosis of CJD had negative neural-autoantibody entries but the sporadic subtype was not separately established (44); and Bilavu et al., a previous systematic review. Neither Maat nor van Steenhoven satisfies the eligibility rule stated in Section 2.2 requiring an extractable sporadic-CJD antibody numerator and denominator. Van Steenhoven et al. is not a suspected-CJD cohort but a prospective RPD cohort and is labeled as external RPD context throughout; antibody results were not reported separately for the 17 patients with a final diagnosis of CJD and the sporadic subtype was not specified, so no independent sCJD numerator and denominator can be extracted. None of the four contributes to any counted denominator. The evidence base was dominated by single case reports and small series (8–38); references 28 and 36 are the Chen and Mackay multi-patient reports and are discussed separately. These reports were valuable for identifying the spectrum of antibodies, false-positive results, dual-pathology scenarios and immunotherapy responses, but they do not provide an unbiased sampling frame for frequency estimation, and only the six multi-patient datasets contributed to the quantitative frequency synthesis: Gurram et al. (6), Lazar et al. (39), Ong et al. (40), Rossi et al. (5), Chen et al. (28) and Grau-Rivera et al. (41). Their key quantitative features, including specimen and the basis of the sCJD diagnosis, are shown in **Table 1**.

The seven reports added by the supplementary search included two with positive neuronal-antibody findings and five with named negative results (45–51). The positive reports described a low-positive glycine-receptor signal in autopsy-confirmed sCJD and serum anti-Yo in clinically diagnosed sCJD with coexisting lung adenocarcinoma; neither establishes a causal autoimmune mechanism. One negative report concerned autopsy-confirmed sporadic fatal insomnia and is retained as explicitly labeled sporadic prion-subtype context. None contributes to a frequency denominator. **Supplementary Table 6** provides diagnostic, specimen and antibody details. Review of previously included reports also confirmed serum GlyRα1 IgG at 1:30 with negative CSF in Wu et al. (9), and negative serum NMDAR and named onconeural testing in Molina and Fekete (37).

### 3.3 Primary outcome: neuronal-antibody frequency in sCJD

Across the six independent multi-patient datasets, 241 patients with a final clinical or pathological diagnosis of sCJD underwent the reported antibody testing for the prespecified neuronal-surface antibodies; the panels and assays differed by dataset (**Table 1**). Four patients were positive, all of them in Rossi et al. (4/82): three had an identified neuronal-surface antigen (CASPR2 in three, of whom two were also glycine-receptor positive and one also NMDAR positive), and the fourth had an isolated low-level VGKC-complex result without LGI1 or CASPR2 specificity. All were low-level serum results. That report included 42 postmortem-confirmed and 40 clinically diagnosed patients, and the diagnostic certainty of the four antibody-positive patients was not reported separately. The remaining five datasets, comprising 49 pathologically confirmed cases (Grau-Rivera), 79 cases (Lazar), 21 cases (Gurram), 8 cases (Chen) and 2 autopsy-confirmed cases (Ong), reported none.

The aggregate proportion was 4 of 241 (1.66%; exact 95% CI 0.45 to 4.19). The random-intercept GLMM gave a pooled estimate of 0.36% (profile 95% CI lower bound <0.01%, upper bound 3.38), and the beta-binomial model 1.21% (profile 95% CI 0.07 to 19.67). These summaries differ in estimand and distributional assumptions (Section 2.7); their divergence, from 0.36% to 1.66% for the point estimate and from 3.4% to 19.7% for the upper confidence bound, and the width of the intervals limit confidence in any single transferable frequency estimate. The descriptive heterogeneity statistics were Q = 4.17 on 5 degrees of freedom with I-squared = 0.0%; with five zero-event datasets these statistics are uninformative and are not interpreted as evidence of homogeneity (**Figure 2**).

**Figure 2:**
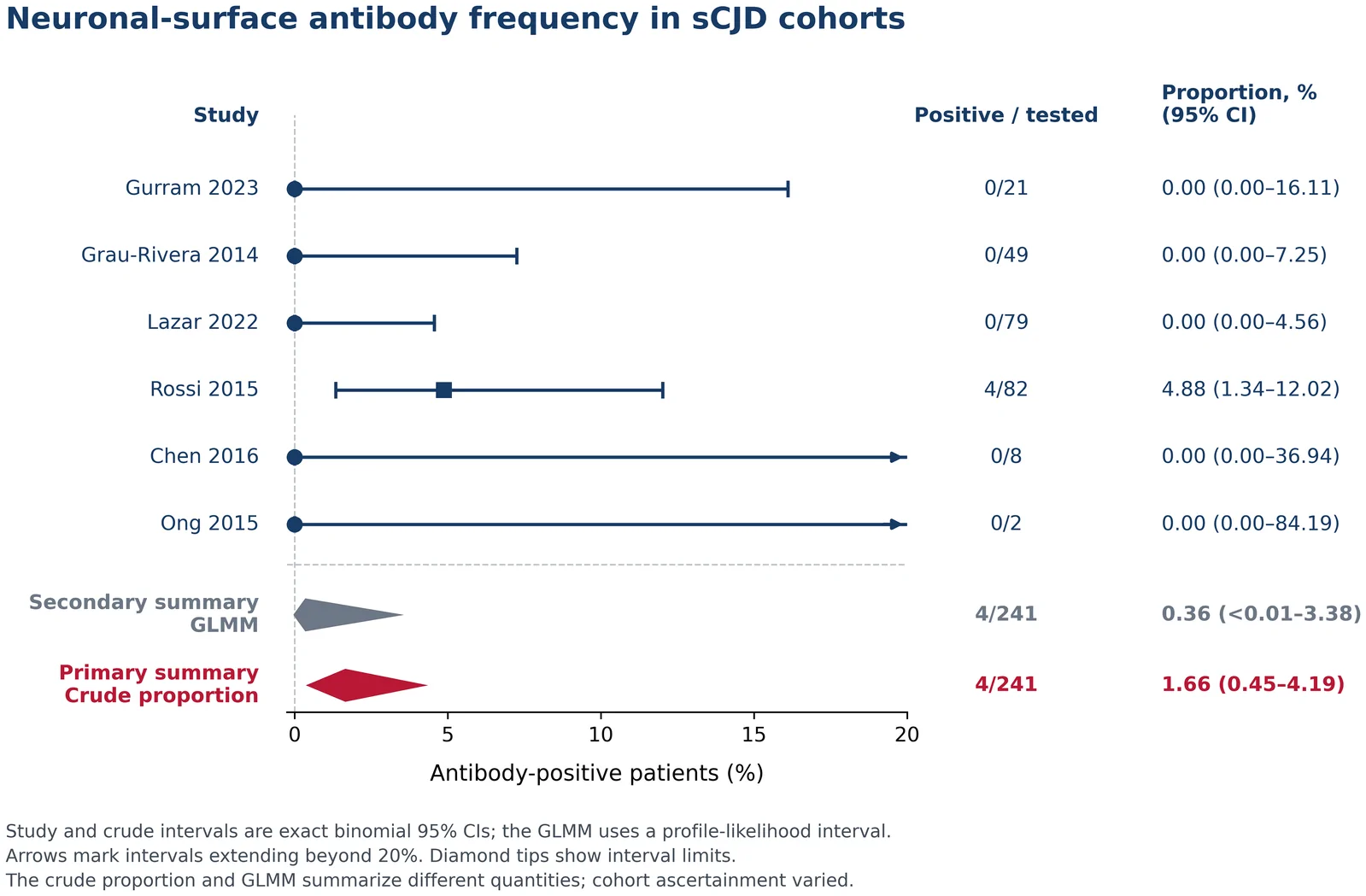
Forest plot for the primary frequency analysis. Study-level intervals are exact Clopper-Pearson binomial 95% confidence intervals; intervals extending beyond the plotted range are shown with an arrow. Marker shape distinguishes datasets with at least one positive result (square) from zero-event datasets (circle). The red diamond is the aggregate (crude) proportion with its exact binomial interval, reported as a descriptive primary summary; the grey diamond is the random-intercept logistic-normal GLMM estimate with its profile-likelihood interval. The two summaries differ in estimand and assumptions; the crude interval does not account for between-study heterogeneity, and the sparse data limit interpretation of the random-effects estimate.

Pathologically confirmed patients. No neuronal-surface antibody was detected among the 51 pathologically confirmed patients from the two non-overlapping datasets in which antibody status could be directly linked to pathological confirmation (Grau-Rivera 0/49 definite CJD on a comprehensive CSF neuronal-surface panel; Ong 0/2 on legacy VGKC-complex testing), giving an exact upper 95% confidence bound of 7.0%. Rossi et al. included an additional 42 postmortem-confirmed patients among the 82 systematically retested, for whom the positive and negative antibody results could not be cross-tabulated with pathological confirmation, so the antibody numerator among those 42 is unknown and they cannot be counted as antibody-negative. Nor do the 93 patients across these three reports represent all pathologically confirmed patients in the evidence base: the Lazar parent cohort of 93 also included 33 pathologically proven patients, for whom a pathology-by-antibody-testing cross-tabulation was not reported (39). Separately, Mackay et al. described low-titer serum NMDAR antibodies (1:80, CSF negative) in 2 of 6 tested patients from a pathologically confirmed sCJD series that was excluded from quantitative synthesis because overlap with the Rossi population could not be excluded (Section 2.5). The 0/51 result therefore bounds, and does not exclude, antibody positivity in neuropathologically confirmed disease.

### 3.4 Sensitivity analyses and antibody specificity

Two prespecified and three *post hoc* sensitivity analyses were performed. First, restricting positivity to antibodies with an identified neuronal-surface antigen, and therefore excluding the isolated VGKC-complex result, gave 3 of 241 (1.24%; exact 95% CI 0.26 to 3.59; GLMM 0.51%, profile 95% CI lower bound <0.01%, upper bound 3.03). Second, restricting to datasets with at least 10 tested patients (excluding Chen, n = 8, and Ong, n = 2) left the estimate essentially unchanged at 4 of 231 (1.73%; 95% CI 0.47 to 4.37).

Third, in a *post hoc* analysis by study quality, excluding the two datasets rated at high risk of bias (Lazar 2022, n = 79, and Chen 2016, n = 8), both of which were zero-event, gave 4 of 154 (2.60%; exact 95% CI 0.71 to 6.52; GLMM 1.75%, profile 95% CI lower bound <0.01%, upper bound 7.35); with the surface-antigen restriction applied as well, 3 of 154 (1.95%; 0.40 to 5.59). Restricting instead to the single dataset rated at low risk of bias (Gurram 2023) gave 0 of 21 (exact upper 95% bound 16.1%). The aggregate estimate therefore rises when lower-quality datasets are removed, because those datasets contributed only to the denominator, which illustrates that the exact numerical value depends on denominator composition even though the observed proportions remain low under every specification. Fourth, excluding the Gurram dataset, whose 21 tested patients could not be separated by diagnostic certainty, gave 4 of 220 (1.82%; exact 95% CI 0.50 to 4.59); this addresses that cohort’s inclusion but cannot remove diagnostic or selection uncertainty in the remaining datasets. Fifth, the beta-binomial model reported in Section 3.3 examined sensitivity to model specification.

Finally, an exploratory analysis counting any reported neural or neuromuscular seroreactivity regardless of antigen target gave 13 of 233 (5.58%; exact 95% CI 3.00 to 9.35; GLMM 2.53%, profile 95% CI 0.01 to 9.13). This analysis uses the serum denominator for Lazar et al. (9/71) rather than the cerebrospinal fluid denominator used in the primary analysis, because the additional reactivities were detected in serum only; the total denominator is therefore 233 rather than 241. The additional positives under that exploratory definition were all in Lazar et al. and comprised five low-level GAD65 reactivities, two voltage-gated calcium channel results, one acetylcholine-receptor result and one striated-muscle antibody. The GAD65 titers were very low (n = 5; median 0.06 nmol/L, range 0.05 to 0.13; laboratory expected 0.02 nmol/L or below), two to three orders of magnitude below the serum threshold of >20 nmol/L used to define high-titer GAD65 antibodies in reference cohorts (Section 4.4).

### 3.5 Diagnostic significance in suspected CJD

The suspected-CJD literature addressed a different clinical question and was therefore not merged with the sCJD frequency denominator. In the prospective suspected-CJD arm of Grau-Rivera et al., 6 of 346 patients (1.7%) had a CNS antibody-associated disorder; the six target antigens were CASPR2, LGI1, NMDAR, AQP4, Tr (DNER) and one unidentified neuronal-surface antigen. Five met this review’s neuronal-antibody definition; the AQP4 case was excluded because AQP4 is astrocytic rather than neuronal, and each corresponded to a defined, treatable syndrome distinct from prion disease. None of the six fulfilled possible or probable CJD criteria, and all improved or stabilized with diagnosis-specific treatment (41). A large autopsy series (Maat et al., 384 patients) similarly tested for neuronal antibodies but did not report an extractable sCJD-specific numerator and denominator, and so, like the suspected-CJD datasets, could not contribute to the primary estimate; it is summarized with the other suspected/mixed-population data in **Table 2** (42). As external RPD context rather than suspected-CJD evidence, a recent prospective multicenter cohort of 147 patients with rapidly progressive dementia found autoimmune encephalitis to be the single most common treatment-responsive cause (58/147, 39%), substantially exceeding the proportion ultimately attributable to prion disease (17/147, 12%), and reported that movement disorders emerge early in autoimmune encephalitis but late in CJD, a potentially useful early discriminator (43).

**Table 2:** Key evidence on diagnostic significance in suspected CJD, with external RPD context.

Study	Population	Key finding	Antibody-related result	Interpretation
Grau-Rivera 2014 (41)	346 prospectively referred suspected CJD	6 CNS antibody-associated disorders	5/346 met this review’s neuronal-antibody definition (1.4%); 6/346 including AQP4	Treatable mimics; none met possible/probable CJD criteria
Lazar 2022 (39)	11 prospectively enrolled mimics vs 93 CJD cases	All 11 mimics met probable-CJD criteria; 6 had AE	Neural-specific antibodies in 5/9 serum and 5/10 CSF of mimics	Autoimmune disease can satisfy clinical CJD criteria
Rossi 2015 (5)	Referral experience; denominator not reported	3 autoimmune encephalitis patients referred as possible sCJD	High VGKC-complex/LGI1 antibodies	High-titer, phenotype-concordant antibodies favored AE
Maat 2015 (42)	Autopsy series (384) of suspected CJD	Neuronal antibodies tested; no extractable sCJD numerator/denominator	Not poolable	Contextual; excluded from frequency analysis
van Steenhoven 2026 (43) — external RPD context	147 prospective RPD patients (multicenter); not a suspected-CJD cohort	AE most common treatable cause (58/147, 39%); CJD 17/147 (12%)	Movement disorders early in AE, late in CJD	External RPD context supporting antibody testing to detect treatable mimics; antibody results not reported separately for CJD

These heterogeneous referral and case-control populations were not statistically pooled and are reported narratively. Maat 2015 and van Steenhoven 2026 did not contribute to the primary frequency analysis and are not among the six datasets shown in **Table 1**; van Steenhoven 2026 is a prospective RPD cohort rather than a suspected-CJD cohort and is included as external RPD context only. Grau-Rivera 2014, Lazar 2022 and Rossi 2015 appear in both tables because each provided an sCJD subgroup for the frequency analysis in addition to suspected-CJD data reported here. AE, autoimmune encephalitis; RPD, rapidly progressive dementia.

Lazar et al. compared 11 prospectively enrolled mimics, all of whom met clinical criteria for probable CJD, with 93 patients with definite or probable CJD; six of the 11 mimics had autoimmune encephalitis, and neural-specific antibodies were detected in serum (5/9) and CSF (5/10) of the mimics (39). Rossi et al. additionally described three patients initially referred as possible sCJD who instead had autoimmune encephalitis with high-titer LGI1/CASPR2 antibodies (5). These studies consistently support the clinical value of antibody testing in selected suspected-CJD presentations, but their denominators and ascertainment strategies are not sufficiently comparable for a single pooled diagnostic-yield estimate. **Figure 3** summarizes the resulting differential-diagnostic framework.

**Figure 3:**
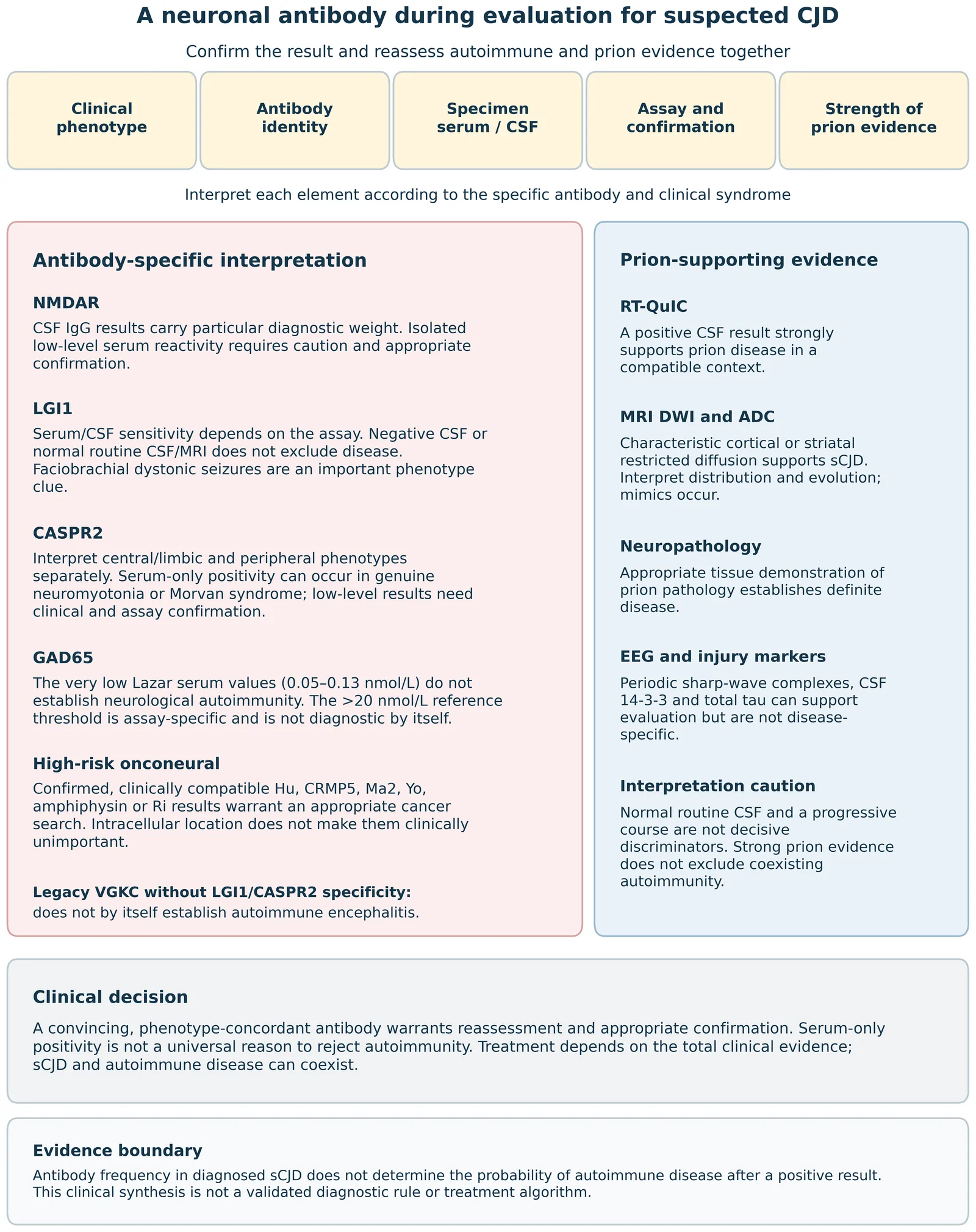
Differential-diagnostic framework for a neuronal antibody detected during the work-up of suspected CJD. Antibody frequency in sCJD and diagnostic yield in suspected CJD answer different questions and should not share a denominator. Interpretation is organized around five elements, clinical phenotype, antibody identity, specimen, assay and confirmation, and strength of prion evidence, rather than a universal hierarchy of titer, CSF positivity and inflammation. Features supporting autoimmune encephalitis are shown for each antibody group (NMDAR, LGI1, CASPR2, very low-level GAD65, and high-risk onconeural antibodies), with the compartment of greatest diagnostic reliability indicated where it differs; features supporting prion disease, in particular a positive RT-QuIC and a characteristic diffusion-restriction pattern, are set opposite. The two diagnoses are not invariably mutually exclusive, and treatment decisions rest on the clinical syndrome and the total evidence.

### 3.6 Risk of bias and certainty of evidence

Formal JBI appraisal was conducted independently in duplicate (NQ and ES) using tools matched to study design, with a third reviewer (AA) adjudicating all item-level disagreements and recording a written rationale for each. For the five datasets appraised together, reviewers agreed on 33 of 50 items (raw agreement 0.66) and all 17 disagreements were adjudicated. Chen 2016 was appraised separately in the same format after full-text verification; reviewers agreed on 5 of 10 items and all 5 disagreements were adjudicated. Overall ratings were: Gurram 2023 low; Grau-Rivera 2014, Rossi 2015 and Ong 2015 moderate; and Lazar 2022 and Chen 2016 high (**Figure 4**). These design-matched ratings do not eliminate selection bias or incomplete antibody ascertainment when estimating frequency. Viewed specifically from that standpoint, two features not captured by those ratings are relevant: antibody testing in Gurram et al. covered 21 of 25 patients and the diagnostic-certainty distribution of the tested subset is not separately available, which is a coverage and selection limitation despite the low design-matched rating; and only the 82 retrieved sera in Rossi et al. underwent uniform panel testing, which is why that subset rather than the full clinical experience was used. The quality-based sensitivity analyses in Section 3.4 address the influence of these features on the estimate directly.

**Figure 4:**
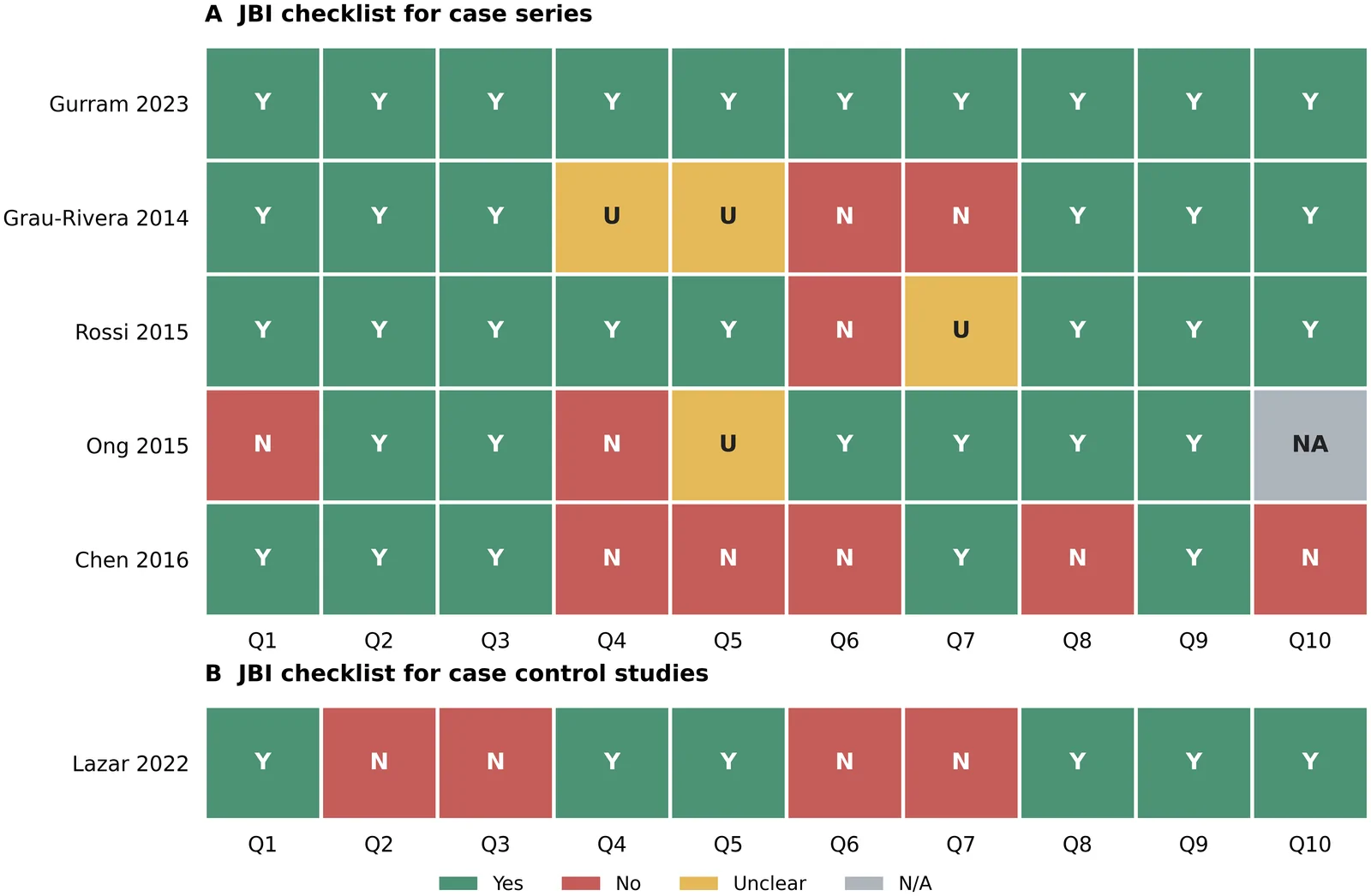
Item-level JBI critical appraisal for the six multi-patient datasets in the primary analysis. Judgments are the final adjudicated ratings: items on which the two reviewers agreed are the consensus judgment, and disagreements are the third reviewer’s adjudicated judgment. Overall: Gurram low; Grau-Rivera, Rossi and Ong moderate; Lazar and Chen high. The figure is presented as two panels because two instruments were used: Panel **(A)** shows the ten items of the JBI Checklist for Case Series for Gurram, Grau-Rivera, Rossi, Chen and Ong, and Panel **(B)** the ten items of the JBI Checklist for Case Control Studies for Lazar 2022. The two item sets are not equivalent and are mapped side by side in **Supplementary Table 4**. Overall risk-of-bias ratings are stated above and in **Supplementary Table 3**; item wording is given in **Supplementary Table 4**. Y = Yes, N = No, U = Unclear, NA = not applicable; symbols as well as colour encode each judgment. The six-dataset matrix contains 60 items, comprising 38 reviewer agreements and 22 adjudicated disagreements.

## 4 Discussion

### 4.1 Principal findings

This systematic review separates two questions that have frequently been mixed in the CJD-antibody literature. Among patients with a final clinical or pathological diagnosis of sporadic CJD who underwent standardized testing, neuronal-surface antibodies were uncommon: an aggregate proportion of 1.66% (4 of 241), with all four positive results arising from a single dataset, and model-based summaries ranging from 0.36% to 1.21% with upper confidence bounds from 3.4% to 19.7%. No positive result was recorded among the 51 pathologically confirmed patients from the two non-overlapping datasets in which antibody status could be linked directly to pathological confirmation, although the Rossi cohort contained a further 42 postmortem-confirmed patients whose antibody status could not be linked individually to pathological status, and Mackay et al. reported low-titer serum NMDAR reactivity in 2 of 6 tested patients from a pathologically confirmed series that could not be included because of possible overlap. Of the four positives, three had an identified neuronal-surface antigen and the fourth was an isolated VGKC-complex result; all were low-level serum reactivities of limited or uncertain clinical specificity, and their individual pathological status and detailed phenotype are not available. In contrast, antibody testing within the diagnostic work-up of suspected CJD occasionally identified treatable autoimmune disease. These are complementary findings, not competing estimates.

The primary result should not be interpreted as evidence that a positive antibody rules out CJD, nor as evidence that autoimmune disease is probable whenever an antibody is found. The quantity estimated here is approximately the probability of a positive neuronal-surface antibody given a final diagnosis of sCJD. The clinically relevant quantity after a positive result is different: the probability of autoimmune encephalitis or another immune-mediated disorder given the antibody, the phenotype and the results of the CJD investigations. The latter depends on the pre-test probability of autoimmune disease in the referral population, the antibody- and assay-specific specificity, the biological compartment tested, and the strength of the evidence for prion disease, none of which is supplied by a frequency estimate in sCJD. Because no contemporaneous comparator arm was available, this review cannot estimate a positive predictive value (Section 4.3). The frequency data alone cannot distinguish incidental reactivity, a secondary immune response and coexisting autoimmune disease. What they do establish is narrower: antibody positivity is not a common accompaniment of sCJD in tested cohorts, so a positive result cannot be dismissed as an expected finding of prion disease and warrants confirmation and clinical reassessment, with the weight given to it determined by the antibody-specific considerations in Section 4.4 rather than by inversion of the frequency estimate. Isolated antibody-positive cases with neuropathologically or biomarker-confirmed CJD are documented, including NMDAR-, LGI1-, CASPR2-, GAD65- and onconeural-antibody reports (14, 16, 24, 29, 32–36), although many reported titers were low, serum-only or not reproducible on confirmatory testing; and Rossi et al. demonstrated low-level neuronal antibodies in a small minority of patients who ultimately had sCJD (5). The two diagnoses are, moreover, not invariably mutually exclusive: Kim et al. described a patient with clinically active LGI1 autoimmunity, including immunotherapy-responsive faciobrachial dystonic seizures, who subsequently developed rapidly progressive dementia confirmed as sCJD at autopsy (29), and Seth et al. reported a similar sequence in a patient meeting probable-CJD criteria (20). The four positive results in the primary analysis should also be interpreted against current assay standards: isolated VGKC-complex positivity without LGI1 or CASPR2 specificity does not by itself support autoimmune encephalitis (52), while low-level serum CASPR2 reactivity has limited diagnostic specificity and should be interpreted in clinical context (53, 54). Accordingly, the low-level CASPR2 findings reported by Rossi et al. do not, by themselves, support concurrent autoimmune encephalitis. Because only six datasets and four positive patients underpin the primary estimate, its precision remains limited.

The suspected-CJD data reinforce this point. Grau-Rivera et al. prospectively detected treatable antibody-associated disorders in a small but clinically meaningful fraction of referrals, and all six antibody-associated patients improved or stabilized (41). Lazar et al. showed that autoimmune and other treatable disorders can satisfy clinical criteria for probable CJD (39). Maat et al., summarized in **Table 2** and in Section 3.5, adds external context: 22 of 384 suspected-CJD autopsies showed autoimmune encephalitis (42). These diagnostic-yield observations support antibody testing in clinically selected presentations; the weight of a positive finding depends on its antibody-specific specificity and phenotype concordance and cannot be quantified from the sCJD frequency estimate alone.

Our findings also refine the interpretation of the recent review by Bilavu et al. (3). That review appropriately catalogued antibody-positive CJD cases and highlighted the possibility of secondary or incidental immune responses. The present analysis adds the denominator that a case-only synthesis cannot provide: when antibody-negative tested patients are included and overlapping reports are removed, the frequency in patients with a final diagnosis of sCJD is low. This denominator-based perspective explains why an extensive case literature can coexist with a low observed frequency in tested cohorts. To our knowledge this is the first denominator-based frequency estimate of neuronal antibodies in sCJD, as distinct from a catalogue of antibody-positive cases.

### 4.2 Methodological considerations

Several methodological choices were important. We did not combine suspected-CJD referrals with patients with a final diagnosis of sCJD because those denominators answer different clinical questions. We did not add overlapping testing rounds or publications. We did not treat single case reports as frequency data. We report the aggregate proportion as a descriptive count summary because, with four events in six datasets, a random-effects model is weakly identified; but the sparse-data limitations of random-effects models do not make the aggregate proportion a better meta-analytic estimator. It implicitly treats all 241 patients as one binomial sample despite substantial differences among the datasets in source population, selection, specimen, antibody panel and assay method, and the GLMM, which retains study-level binomial structure, is the more appropriate model in principle even where it is imprecise. We therefore present the aggregate proportion, the GLMM and the beta-binomial model together, distinguishing their estimands and interpreting their divergence cautiously. Quantitative combination of these six datasets is justified only in the limited sense that each contributes a tested denominator of patients with a final diagnosis of sCJD, subject to the Gurram diagnostic-certainty limitation stated above, and a numerator on the reported neuronal-surface assays, whose panel coverage and sensitivity nevertheless differ materially; it is not justified as an estimate of a single underlying prevalence, and we do not present it as one. We used a logistic-normal GLMM rather than a Freeman-Tukey double-arcsine analysis, because back-transformation of the latter can be misleading in meta-analyses of rare single proportions (7), and we do not interpret I-squared = 0 as homogeneity, since with five zero-event datasets the statistic has essentially no power. Risk of bias bears on the primary result in a specific direction: the only neuronal-surface antibodies arose from a single moderate-risk dataset (Rossi) and were low-level, whereas the largest pathologically confirmed dataset (Grau-Rivera, 0/49) and the only low-risk dataset (Gurram, 0/21) detected none, and the additional low-level reactivities in the exploratory analysis derive entirely from a high-risk dataset and involve targets outside the primary encephalitis-antibody definition. Because both high-risk datasets were zero-event, excluding them raises the aggregate proportion to 2.60% (4/154) while leaving the qualitative conclusion unchanged (Section 3.4).

### 4.3 Limitations and future directions

Limitations remain substantial. Only six independent multi-patient datasets contributed to the primary synthesis, antibody panels and assay methods were heterogeneous, serum and cerebrospinal fluid were not uniformly tested together, and several cohorts used clinically selected rather than systematic antibody testing. The antibody block of the original database search was narrower than the eligibility definition; the supplementary search described in Section 2.3 addressed this and identified seven additional qualitative reports but no additional frequency dataset. One potentially relevant report (Liu et al.) could not be obtained as a full text and its antibody results remain unassessed. The estimate is sensitive to how positivity is defined: the primary outcome counts positivity on the neuronal-surface/synaptic assays used in the source studies, restricting it to antibodies with an identified neuronal-surface antigen lowers the estimate, and the exploratory definition counting any reported neural or neuromuscular seroreactivity raises it roughly threefold. That exploratory figure is driven by very low-level GAD65, voltage-gated calcium channel, acetylcholine-receptor and striated-muscle results, all of which fall outside the primary neuronal-surface encephalitis-antibody definition used here: GAD65 is intracellular, VGCC is a neuronal membrane channel outside that definition, muscle AChR is a neuromuscular-junction target, and striated-muscle antibodies are not neuronal antibodies; low-titer GAD65 reactivity in particular can occur outside neurological autoimmunity and is not diagnostic in isolation. We therefore regard the specificity-restricted figure as the most clinically meaningful and the exploratory figure as an illustration of the effect of a broader antibody definition rather than an estimate of neuronal autoimmunity. The estimate is also sensitive to model specification and to denominator composition, as Sections 3.3 and 3.4 show. Individual antibody-positive pathologically confirmed cases have been reported in the wider literature, and 42 postmortem-confirmed patients in the Rossi cohort could not be assigned an antibody status, so the absence of positives among the 51 pathologically confirmed patients here bounds rather than excludes coexistence. The suspected-CJD literature is even more heterogeneous, preventing a defensible pooled diagnostic-yield estimate. The absence of a contemporaneous comparator arm from non-prion rapidly progressive dementia means that these data describe observed antibody coexistence without establishing whether it is incidental, and do not yield a formal positive predictive value for a treatable mimic; establishing that value is the logical next study.

Future studies should prospectively enroll consecutive patients with rapidly progressive dementia, apply the same predefined serum and CSF neuronal-antibody panel to all participants, use modern sCJD criteria with RT-QuIC and neuropathologic confirmation where available, and report results separately for (1) patients ultimately confirmed as sCJD and (2) antibody-associated mimics. Such studies would allow direct estimation of both coexistence frequency and diagnostic yield without the denominator ambiguity that characterizes the current literature.

### 4.4 Antibody-specific interpretation and clinical implications

For the clinician facing a patient with rapidly progressive dementia and a positive neuronal antibody, these data support a graded, antibody-specific interpretation rather than a binary one. A uniform hierarchy based on titer, CSF positivity and inflammatory CSF/MRI findings cannot be applied across antibodies, because the diagnostic weight of specimen, titer and ancillary findings differs by antigen.

For NMDAR antibodies, CSF testing carries particular diagnostic weight: CSF is more reliable than serum, and isolated low-level serum reactivity without orthogonal confirmation requires caution (55). The low-titer serum-only NMDAR results described by Mackay et al. in pathologically confirmed sCJD, with negative CSF, are consistent with this. For LGI1 antibodies the reverse holds in important respects, although relative serum and CSF sensitivity depends on assay methodology: in a large reference-laboratory series CSF testing detected 24 of 38 (63%) seropositive patients (56), and routine CSF and MRI can be normal in anti-LGI1 encephalitis (57). A general rule that CSF concordance or inflammatory CSF/MRI findings substantially increase diagnostic weight would therefore under-recognize true LGI1 disease, for which faciobrachial dystonic seizures provide an important phenotype clue (20, 29). CASPR2 antibodies likewise require phenotype-specific interpretation: CSF positivity is particularly associated with central and limbic phenotypes, whereas genuine neuromyotonia or Morvan syndrome may occur with serum-only antibodies, and low-level serum reactivity has limited specificity (53, 54). For GAD65, the reactivities detected in Lazar et al. (0.05 to 0.13 nmol/L) lie two to three orders of magnitude below the serum threshold of >20 nmol/L used to define high-titer GAD65 antibodies, which are themselves suggestive of but not pathognomonic for GAD65 neurological autoimmunity (58); such very low-level results have limited neurological significance. That statement is specific to very low-level GAD65 and should not be extended to the classical onconeural antibodies (Hu/ANNA-1, CV2/CRMP5, Ma2, Yo, amphiphysin, Ri/ANNA-2). A high-risk antibody directed against an intracellular antigen may not itself be pathogenic and may predict limited immunotherapy responsiveness, but a confirmed, clinically compatible result remains a meaningful biomarker of paraneoplastic neurological disease and warrants an appropriate malignancy search; current PNS-Care criteria classify these antibodies explicitly according to cancer risk (59). An isolated low-titer serum result against an intracellular antigen in a patient with strong prion evidence should therefore be interpreted according to which antigen is involved: a very low-level GAD65 result alone does not establish GAD65 neurological autoimmunity, whereas a high-risk onconeural antibody requires the appropriate malignancy work-up regardless of the CJD investigations.

The framework in **Figure 3** accordingly organizes interpretation around five elements rather than a universal hierarchy: the clinical phenotype and its concordance with the recognized syndrome for that antibody; the antibody identity; the specimen and, where relevant, the compartment in which the antibody is most reliably detected; assay type and orthogonal confirmation; and the strength of the evidence for prion disease, in particular a positive RT-QuIC and a characteristic diffusion-restriction pattern, which are set opposite the autoimmune features so that the framework functions as a true differential-diagnostic tool.

Two practical conclusions follow. First, a convincing neuronal-surface antibody found during a CJD work-up should prompt reassessment of both the autoimmune and the prion evidence, with confirmatory antibody testing in the appropriate compartment, rather than attribution to the prion disease. Second, because the two diseases are not invariably mutually exclusive (20, 29), reassessment does not by itself settle the treatment question. Treatment decisions should depend on the clinical syndrome and the totality of the evidence: where the phenotype supports autoimmune encephalitis and the antibody finding is convincing for that antigen, immunotherapy may be warranted in accordance with established autoimmune-encephalitis frameworks while definitive prion investigations are pending, even when prion disease has not been excluded; a low-level or unconfirmed result alone does not establish an indication for immunotherapy, which in genuine prion disease carries cost and risk without benefit, and neither serum-only positivity nor strong prion evidence should be applied as a universal exclusion rule. This review estimates antibody frequency and diagnostic significance and does not itself evaluate the risk-benefit balance of empiric immunotherapy in any individual case.

## 5 Conclusions

Neuronal-surface antibodies were uncommon in patients with a final clinical or pathological diagnosis of sporadic CJD: the aggregate proportion was 4 of 241 (1.66%), with four low-level serum positives from one cohort, falling to 1.24% when positivity was restricted to antibodies with an identified neuronal-surface antigen and rising to 2.60% when datasets at high risk of bias were excluded; model-based summaries were 0.36% and 1.21% with wide intervals. The estimate therefore changed with antibody definition, denominator composition and model assumptions. No antibody was detected among the 51 pathologically confirmed patients in the two included datasets in which antibody status could be linked directly to pathology, but this selected subset does not establish absence of antibodies in pathologically confirmed disease: other confirmed patients could not be linked individually to testing, an overlapping series excluded from synthesis reported low-titer serum positives in confirmed patients, and antibody-positive confirmed cases have been reported. These data do not estimate the probability of autoimmune encephalitis after a positive result or determine immunotherapy benefit. A convincing antibody finding during evaluation for suspected CJD warrants confirmation and reassessment in the context of antibody identity, specimen, phenotype and prion evidence, with treatment guided by the clinical syndrome and the total evidence; autoimmune encephalitis and sCJD can coexist. Frequency in sCJD and diagnostic yield in suspected CJD should remain separate outcomes in both research and clinical interpretation.

## Data Availability

The original contributions presented in the study are included in the article/**Supplementary Material**. Further inquiries can be directed to the corresponding author.

## Supplementary material

Supplementary Data Sheet 1 The complete search strategy for each database for the original search of 12 July 2026 (**Supplementary Table 1**); the reconciled table of full-text decisions with assigned codes (**Supplementary Table 2**); the JBI critical appraisal (**Supplementary Table 3**) and the mapping of the two JBI instruments (**Supplementary Table 4**); the denominator adjudication (**Supplementary Table 5**); the qualitative synthesis of reports added by the supplementary search (**Supplementary Table 6**); the **Supplementary Methods** on certainty of evidence and statistical synthesis, including the beta-binomial specification; and the PRISMA 2020 checklist.

Supplementary Data Sheet 2 The complete Python analysis script (cjd_meta_analysis.py), which implements the generalized linear mixed model and the beta-binomial model, with the study-level counts entering the primary and sensitivity analyses, the validation cases used to verify the implementation of the generalized linear mixed model, the eligibility-sensitivity script and results, and the analysis verification log. **Supplementary Data 2**: the executed supplementary-search strategies of 13 September 2026 for PubMed, Embase.com and CENTRAL, raw database exports, deduplication audit, record-level reconciliation with the original screening set, the reconciled set of records entering screening, and the 43-report register.
