# Supplementary material for: Neuronal-surface antibodies are uncommon in patients with a final diagnosis of sporadic Creutzfeldt-Jakob disease: a systematic review and meta-analysis
Source: Front Immunol. 2026 Oct 6;17:1966990. doi: 10.3389/fimmu.2026.1966990 (PMC13680256; doi:10.3389/fimmu.2026.1966990)
Supplement: Supplementary file 1 [file DataSheet1.pdf]

Supplementary Material 1

Neuronal-surface antibodies are uncommon in patients with a final diagnosis of sporadic Creutzfeldt-Jakob disease: a systematic review and meta-analysis

Qadi N, Aldakheel A, Shosha E

PROSPERO CRD420261458659

Supplementary Table 1. Full electronic search strategies

For the original review, databases were searched from inception to 12 July 2026 with no date, language or study-design restrictions. Two concept blocks were combined with AND: (1) Creutzfeldt-Jakob disease, prion disease, or rapidly progressive dementia; and (2) neuronal antibodies or autoimmune encephalitis.

Database (interface)	Search string
PubMed / MEDLINE	("Creutzfeldt-Jakob Syndrome"[Mesh] OR "prion disease*"[tiab] OR "Creutzfeldt-Jakob"[tiab] OR "rapidly progressive dementia"[tiab]) AND ("neuronal antibod*"[tiab] OR "autoimmune encephalitis"[tiab] OR "neuronal surface antib*"[tiab] OR NMDAR[tiab] OR LGI1[tiab] OR CASPR2[tiab] OR "anti-neuronal"[tiab])
Embase (Elsevier)	('Creutzfeldt Jakob disease'/exp OR 'prion disease'/exp OR 'rapidly progressive dementia':ab,ti) AND ('neuronal antibody':ab,ti OR 'autoimmune encephalitis'/exp OR 'neuronal surface antibody':ab,ti OR nmdar:ab,ti OR lgi1:ab,ti OR caspr2:ab,ti)
CENTRAL (Cochrane)	("Creutzfeldt-Jakob" OR "prion disease" OR "rapidly progressive dementia") AND ("neuronal antibod*" OR "autoimmune encephalitis")

Original search: PubMed 212; Embase 678; CENTRAL 0; total 890, with 692 after deduplication. The expanded search retrieved 1,237 records. Publication-level deduplication removed 248 records, leaving 989 unique records; 652 matched publications in the original screening set and 337 were additional records. Assessment of the additional records excluded 330 and retained seven reports for qualitative synthesis. No additional primary frequency dataset was identified.

Supplementary Table 2. Reconciliation of original eligibility decisions

Of 229 reports sought for retrieval, 228 were retrieved and assessed and one was not retrieved. The historical log contained 40 inclusions and 189 exclusions. Reconciliation moved one record from an exclusion category to not retrieved and removed four originally retained reports: one conference abstract under E5 and three reports without a named eligible neuronal-antibody result under E8. Final accounting: 36 retained, 192 excluded and 1 not retrieved (total 229). Codes below refer to this reconciled table; historical workbook codes are preserved in the source records.

Exclusion category	Code	n
Non-sporadic prion disease (familial, genetic, iatrogenic, variant)	E1	77
No extractable sporadic-CJD antibody numerator or denominator	E2	67
Non-neuronal antibody only (e.g. thyroid, neurofilament, ganglioside)	E3	14
Autoimmune-encephalitis population without a defined CJD denominator	E4	10
Other (reviews, basic science, non-primary reports and conference-only reports)	E5	11
Case report without individual-level data	E6	8
Duplicate records	E7	2
No named eligible neuronal-antibody outcome (revision-stage removals)	E8	3
Total excluded		192

Reports removed from the retained set during revision (n = 4)

Report	Year	Reason under the Section 2.2 rule
Suo M, Irani N, Leong W, Lock J, O'Connor K. BMJ Neurol Open 5(Suppl 1):A43	2023	E5: Conference abstract without a full report
Ng M, Westover MB, Cole AJ. Treating seizures in Creutzfeldt-Jakob disease. Epilepsy Behav Case Rep 2:75-79	2014	E8: No eligible neuronal target named with its result
Čiauškaitė J, Puleikytė I, Jesmanas S, et al. Clin Case Rep 9:e04699	2021	E8: No eligible neuronal target named with its result
Pimentel GA, Guimarães TG, Silva GD, Scaff M. Front Neurol 13:731369	2022	E8: No eligible neuronal target named with its result

Not retrieved (n = 1)

Liu Y, Jin W, Zang W, Li S, Zhang J. Clinical features in 8 patients of sporadic Creutzfeldt-Jakob disease with positive real-time quaking-induced conversion [in Chinese]. Chin J Neurol (2022) 55:1252-1260. doi: 10.3760/cma.j.cn113694-20220314-00190. The published abstract states that antibodies related to autoimmune encephalitis and paraneoplastic syndrome were collected for the eight RT-QuIC-positive patients but does not report the results or the tested panel; the full report could not be obtained and no antibody result is inferred. In the original master workbook, FT R1 classified record R0225 under historical code E3 (no extractable sporadic-CJD antibody n/N), while FT R2 recorded full text unobtainable. Reconciliation as not retrieved reduces the corresponding category in this table, E2, from 68 to 67; the original decisions remain in the historical log.

The supplementary search contributed 337 additional records: 313 were excluded at title/abstract screening, 24 reports were retrieved and assessed, 17 were excluded at full text and seven were retained for qualitative synthesis. In total, 43 reports were retained.

Supplementary Table 3. JBI critical appraisal of the included datasets

JBI appraisal is complete for all six datasets and 60 checklist items. NQ and ES agreed on 38 items; AA resolved 22 disagreements, comprising 17 across the original five datasets and five for Chen. Final item counts (Yes/No/Unclear/not applicable) were Gurram 10/0/0/0, Grau-Rivera 6/2/2/0, Rossi 8/1/1/0, Ong 6/2/1/1, Lazar 6/4/0/0 and Chen 5/5/0/0. The recorded overall ratings were low for Gurram, moderate for Grau-Rivera, Rossi and Ong, and high for Lazar and Chen. These design-matched ratings do not eliminate selection bias or incomplete antibody ascertainment when estimating frequency.

Dataset	Overall risk of bias	Item summary (Yes / No / Unclear / N/A)	Basis
Gurram 2023	Low	10 / 0 / 0 / 0	Systematic single-centre series; recognised CDC criteria; named antibody panel; all appraisal items met
Grau-Rivera 2014	Moderate	6 / 2 / 2 / 0	Definite-CJD subgroup demographics and clinical detail not reported separately; comprehensive testing and recognised criteria
Lazar 2022	High	6 / 4 / 0 / 0	Case-control ascertainment differed between CJD cases and mimics; confounders not identified or addressed
Rossi 2015	Moderate	8 / 1 / 1 / 0	Short report; incomplete demographic detail; antibody results from systematically retested available sera
Chen 2016	High	5 / 5 / 0 / 0	Only 8 of 68 sporadic-CJD patients underwent antibody testing and the tested subgroup was the non-biopsied, non-consecutive remainder; demographic and outcome reporting for that subgroup incomplete; statistical analysis limited

Dataset	Overall risk of bias	Item summary (Yes / No / Unclear / N/A)	Basis
Ong 2015	Moderate	6 / 2 / 1 / 1	Two-patient series without a sampling frame; autopsy-confirmed; antibody-negative

All 22 disputed JBI items have been adjudicated; the six-study matrix is complete.

Supplementary Table 4. JBI checklist items shown in Figure 4

Item wording is reproduced from the JBI critical appraisal tools (https://jbi.global/critical-appraisal-tools).

Item	JBI Checklist for Case Series (Gurram, Grau-Rivera, Rossi, Chen, Ong)	JBI Checklist for Case Control (Lazar)
Q1	Were there clear criteria for inclusion in the case series?	Were the groups comparable other than the presence of disease in cases or the absence of disease in controls?
Q2	Was the condition measured in a standard, reliable way for all participants included in the case series?	Were cases and controls matched appropriately?
Q3	Were valid methods used for identification of the condition for all participants included in the case series?	Were the same criteria used for identification of cases and controls?
Q4	Did the case series have consecutive inclusion of participants?	Was exposure measured in a standard, valid and reliable way?
Q5	Did the case series have complete inclusion of participants?	Was exposure measured in the same way for cases and controls?
Q6	Was there clear reporting of the demographics of the participants in the study?	Were confounding factors identified?
Q7	Was there clear reporting of clinical information of the participants?	Were strategies to deal with confounding factors stated?
Q8	Were the outcomes or follow-up results of cases clearly reported?	Were outcomes assessed in a standard, valid and reliable way for cases and controls?
Q9	Was there clear reporting of the presenting site(s)/clinic(s) demographic information?	Was the exposure period of interest long enough to be meaningful?
Q10	Was statistical analysis appropriate?	Was appropriate statistical analysis used?

Supplementary Table 5. Denominator adjudication for the primary synthesis

Denominators were required to correspond to standardized ascertainment of the review outcome. Where a study reported more than one testing round, the subset with uniform panel testing was used.

Dataset	Denominator used	Positives	Basis
Gurram 2023	21	0	21 of 25 patients underwent the autoimmune-encephalitis panel; the overlapping 19-patient paraneoplastic panel was retained separately. Diagnostic-certainty distribution of the tested subset was not separately reported.
Grau-Rivera 2014	49	0	All 49 pathologically confirmed cases tested by brain immunohistochemistry plus cell-based assays for LGI1, CASPR2, GlyR and NMDAR.

Dataset	Denominator used	Positives	Basis
Lazar 2022	79	0	The CJD cohort comprised 93 patients (33 definite, 60 probable). No neural-specific autoantibody was identified in any CJD case (serum 0/71, CSF 0/79); nine patients had non-specific serum reactivities outside the primary encephalitis-antibody definition, which contribute only to the exploratory analysis. Panels were reported by specimen and the number of unique patients tested in either compartment was not reported; the larger fully documented single-compartment denominator (79, CSF) was used. Substituting 71 changes the crude estimate from 1.66% to 1.72%.
Rossi 2015	82	4	The 150 patients had clinician-directed, non-uniform testing (VGKC-complex requested in 119, NMDAR in 77, GlyR in 6). Only the 82 retrieved sera underwent the standardized panel (NMDAR, GlyR, VGKC-complex, LGI1, CASPR2). The 150-patient experience is described narratively and was not used as a prevalence denominator.
Ong 2015	2	0	Two autopsy-confirmed VV2 cases; serum VGKC-complex assessed, both negative. Additional serum LGI1 testing was negative in patient 2. RT-QuIC was positive in patient 2 and not performed in patient 1 (Ong, Table, p. 329).
Chen 2016	8	0	Eight non-biopsied sCJD patients under 2009 clinical criteria had serum and CSF tested by indirect immunofluorescence for LGI1, CASPR2, NMDAR, GlyR and intracellular antibodies; all negative. RT-QuIC was not reported. The eight AE mimics are a separate group and do not enter this denominator (Chen, Sections 2.1–2.2, pp. 1–2).
Mackay 2012	not pooled	(2/6)	Review of 100 pathologically confirmed sporadic CJD cases at the UK National CJD Research and Surveillance Unit; six had NMDAR results available, two positive at low titer (1:80 serum), CSF negative in all six. Retained descriptively because patients in the United Kingdom with suspected CJD are commonly referred to one of these two national centres and both series were tested at the same reference laboratory, so patient overlap with the Rossi cohort cannot be excluded.
Total pooled	241	4	Crude proportion 1.66% (exact 95% CI 0.45 to 4.19). The exploratory seroreactivity analysis uses the Lazar serum denominator and therefore totals 233.

Supplementary Methods. Certainty of evidence

Certainty was considered across the domains of risk of bias, imprecision, inconsistency, indirectness and publication bias. This was a domain-based assessment, not a formal GRADE rating; a formal evidence profile was not constructed. Overall confidence in the frequency estimate was low because events were sparse, sampling and diagnostic certainty varied, assay panels differed, and five of six datasets had moderate or high design-matched JBI ratings. These ratings do not capture all sources of bias relevant to estimating frequency.

Supplementary Methods. Statistical synthesis

The primary outcome was positivity on the neuronal-surface/synaptic antibody assays used in the source studies, including legacy voltage-gated potassium channel (VGKC) complex assays. A prespecified specificity-focused sensitivity analysis restricted positivity to antibodies with an identified neuronal-surface antigen.

Primary summary measure. Four events were observed across six datasets, five of which were zero-event. At this event count random-effects models for proportions are weakly identified, and the pooled estimate is sensitive to modelling choices that are not themselves testable with six studies. The crude proportion with an exact Clopper-Pearson binomial interval is therefore reported as the primary summary measure, and the model-based estimate is reported alongside it rather than in place of it.

Model. A random-intercept logistic-normal generalized linear mixed model was fitted. For dataset i with k positives among n tested, k is Binomial(n, expit(mu + b)) with b Normal(0, tau squared). The marginal likelihood was obtained by integrating out the random effect using adaptive quadrature centered and scaled at the mode of each study-specific integrand, and mu and log tau were estimated by maximum likelihood using the Nelder-Mead simplex from five dispersed starting values. No continuity correction was applied in the generalized linear mixed model or in the crude pooled proportion, and zero-event datasets entered the likelihood directly; the descriptive Q and I squared statistics reported below do use a 0.5 continuity correction, to permit finite descriptive study logits. The pooled proportion is expit(mu), which is the median of the random-effects distribution of dataset proportions and need not equal the crude proportion.

Interval estimation. Confidence intervals for mu are profile-likelihood intervals, obtained by inverting the likelihood ratio test at the 95% level against a chi-square distribution with one degree of freedom, with tau re-estimated at each fixed value of mu. Dataset-level intervals are exact Clopper-Pearson binomial intervals. The original 100-node Gauss-Hermite implementation reproduced point estimates but showed unstable lower tails when checked at 300 and 600 nodes. Revised profiling uses adaptive integration and searches local minima over a grid of log tau values, with a zero-variance limit included and log tau bounded above at 5. Endpoints must bracket the likelihood-ratio threshold; failed searches raise an error. Very small positive endpoints are reported as <0.01% rather than exact zero. These are asymptotic intervals and remain uncertain with six sparse datasets.

Heterogeneity. Cochran Q and I squared are reported for description only. They were computed on the logit scale by the DerSimonian-Laird method, using a 0.5 continuity correction for zero-event datasets to permit finite study logits. They are therefore not derived from the generalized linear mixed model described above, are unstable with six datasets and four events, and were not used for inference. The Freeman-Tukey double-arcsine transformation was not used, given documented instability of its back-transformation for proportions near zero with unequal sample sizes.

Sensitivity analyses. Prespecified analyses restricted positivity to identified neuronal-surface antigens and excluded datasets with fewer than 10 tested patients. Selection of the standardized Rossi testing subset was a denominator-adjudication decision used in the primary analysis, not a separate sensitivity result. Post hoc analyses excluded high-risk datasets (Lazar and Chen), restricted analysis to the sole low-risk dataset (Gurram), excluded Gurram because tested-subset diagnostic certainty was inseparable, and used an alternative beta-binomial model. Exploratory analysis counted any reported neural or neuromuscular seroreactivity; Lazar contributes 9/71 serum samples, giving 13/233 overall.

Alternative model. In the post hoc beta-binomial analysis, k_i conditional on p_i follows Binomial(n_i, p_i), and p_i follows Beta(alpha, beta). The marginal likelihood was maximized over positive alpha and beta. The reported mean study probability is alpha/(alpha + beta); its 95% profile-likelihood interval profiles over alpha + beta using a chi-square likelihood-ratio threshold with one degree of freedom. The estimate is 1.21% (0.07–19.67%). This mean probability differs from expit(mu), the median study probability under the logistic-normal GLMM; the difference between model estimates is not a formal uncertainty interval.
Additional crude analyses. Excluding Lazar and Chen gives 4/154 (2.60%; exact 95% CI 0.71–6.52), or 3/154 (1.95%; 0.40–5.59) under the antigen-specific restriction. Restriction to Gurram gives 0/21 (upper endpoint of the two-sided exact 95% CI 16.11%). Excluding Gurram gives 4/220 (1.82%; 0.50–4.59%). These analyses assess sensitivity to dataset composition and do not correct within-study selection bias.

Software and reproducibility. The original analysis used Python 3.12 with NumPy 2.3 and SciPy 1.17. The revised script, cjd_meta_analysis.py (Supplementary Data 1), records the runtime versions used for execution and contains the dataset counts, original and post hoc analyses, and validation checks. Checks cover homogeneous-study maximum-likelihood estimates, the zero-variance binomial identity and agreement with independent Gauss-Hermite quadrature at moderate variance. Output is written to meta_results.json. A computational rerun reproduced the revised outputs and validation checks.

Supplementary Data 1. Analysis script

Provided as a separate file (cjd_meta_analysis.py). Running the script reproduces all pooled estimates, confidence intervals and heterogeneity statistics reported in the main text. Expanded_Search_Strategies.txt and the accompanying search audit are provided as Supplementary Data 2.

Supplementary Table 6. Reports added by the supplementary search

Seven reports contributed qualitative context only. Reference numbers refer to the main manuscript. No report in this table contributes to the primary frequency denominator.

Report	Diagnosis / specimen	Antibody finding and interpretation
Wiels 2018 (45)	33-year-old man; autopsy-confirmed VV1 sCJD; separate serum/CSF titers not reported	GlyR signal at 1:20, not positive at 1:100. No response to immunotherapy; no brain IgG deposits.
Esezoobo 2026 (46)	77-year-old woman; probable Heidenhain-variant sCJD	Named neuronal-antibody testing negative.
Sánchez Pérez 2024 (47)	73-year-old man; probable sCJD, RT-QuIC positive; CSF	NMDAR antibody negative.
Zhong 2023 (48)	58-year-old man; clinically diagnosed sCJD with lung adenocarcinoma; no RT-QuIC or neuropathology	Serum anti-Yo and titin positive; CSF paraneoplastic and serum/CSF AE panels negative. No improvement with IVIg. Coexisting malignancy complicates interpretation.
Ćorić 2012 (49)	57-year-old woman; probable sCJD; serum	Anti-Yo, anti-Hu and anti-Ri negative.
Mehta 2008 (50)	56-year-old woman; autopsy-confirmed sporadic fatal insomnia; CSF	Ma, Ri, Hu, Yo, Ta, CV2 and Zic4 negative. Sporadic prion-subtype context, not a frequency dataset.
Kojima 2013 (51)	66-year-old woman; autopsy-confirmed CJD; serum	Anti-Hu and anti-Purkinje-cell testing reported as normal.

PRISMA 2020 Checklist

Neuronal-surface antibodies are uncommon in patients with a final diagnosis of sporadic Creutzfeldt-Jakob disease: a systematic review and meta-analysis

Qadi N, Aldakheel A, Shosha E

The original search identified 890 records (692 screened after deduplication; 229 sought for retrieval; 228 assessed and one not retrieved). Reconciliation during revision moved one record to not retrieved and removed four originally retained reports (Suo under the full-report criterion; Ng, Čiauškaitė and Pimentel under the named-target criterion), giving 36 retained original reports and 192 full-text exclusions (Supplementary Table 2). The supplementary search (13 September 2026) retrieved 1,237 records; 248 duplicates were removed, leaving 989 unique records, of which 652 matched the original screening set and 337 were additional; 313 were excluded at title/abstract, 24 were assessed at full text, 17 were excluded and seven were retained for qualitative synthesis. No additional frequency dataset was identified. In total 43 reports were retained: six independent multi-patient datasets and 37 qualitative-only reports.

Item #	Section / topic	Checklist item and where it is addressed	Location
	TITLE		
1	Title	Identify the report as a systematic review.	Title
	ABSTRACT		
2	Abstract	See the PRISMA 2020 for Abstracts checklist. Structured abstract with background, methods, results and conclusion; registration reported.	Abstract
	INTRODUCTION		
3	Rationale	Describe the rationale for the review in the context of existing knowledge.	Section 1
4	Objectives	Provide an explicit statement of the objective(s) or question(s) the review addresses (PICOS stated in Section 2.2).	Sections 1, 2.2
	METHODS		
5	Eligibility criteria	Specify the inclusion and exclusion criteria and how studies were grouped for the syntheses. Sections 2.1 and 2.2.	Sections 2.1–2.2
6	Information sources	Specify all databases, registers and other sources searched, and the date each was last searched. Section 2.3; full strategies in Supplementary Table 1.	Section 2.3; Supplementary Table 1; Supplementary Data 2
7	Search strategy	Present the full search strategies for all databases, including any filters and limits used. Supplementary Table 1.	Section 2.3; Supplementary Table 1; Supplementary Data 2
8	Selection process	Specify the methods used to decide whether a study met the inclusion criteria, including how many reviewers screened each record and whether they worked independently. Section 2.4.	Sections 2.4 and 2.8
9	Data collection process	Specify the methods used to collect data from reports, including how many reviewers collected data and whether they worked independently. Section 2.5.	Sections 2.5 and 2.8
10a	Data items (outcomes)	List and define all outcomes for which data were sought. Primary outcome and antibody definition in Section 2.2; denominator adjudication in Section 2.5.	Sections 2.2, 2.5
10b	Data items (other variables)	List and define all other variables for which data were sought (antibody target, specimen, assay, diagnostic certainty). Sections 2.5 and Table 1.	Section 2.5; Table 1
11	Study risk of bias assessment	Specify the methods used to assess risk of bias, including details of the tool(s) used and how many reviewers assessed each study. Section 2.6.	Section 2.6
12	Effect measures	Specify for each outcome the effect measure(s) used in the synthesis or presentation of results. Section 2.7.	Section 2.7
13a	Synthesis methods (eligibility)	Describe the processes used to decide which studies were eligible for each synthesis. Sections 2.2, 2.5 and 2.7.	Sections 2.2, 2.5, 2.7

Item #	Section / topic	Checklist item and where it is addressed	Location
13b	Synthesis methods (preparation)	Describe any methods required to prepare the data for presentation or synthesis. Section 2.7; Supplementary Methods.	Section 2.7; Supplementary Methods
13c	Synthesis methods (tabulation)	Describe any methods used to tabulate or visually display results of individual studies and syntheses. Sections 2.7 and 3.3; Figures 1–4.	Sections 2.7, 3.3; Tables 1–2; Figures 1–4
13d	Synthesis methods (model)	Methods and rationale for synthesis: crude aggregate proportion with exact interval; secondary logistic-normal GLMM; post hoc beta-binomial sensitivity model with a distinct estimand.	Section 2.7; Supplementary Methods
13e	Synthesis methods (heterogeneity)	Describe any methods used to explore possible causes of heterogeneity among study results. Prespecified sensitivity analyses, Section 2.7.	Sections 2.7, 3.4, 3.6
13f	Synthesis methods (sensitivity)	Describe any sensitivity analyses conducted to assess robustness of the synthesised results. Sections 2.7 and 3.4.	Sections 2.7, 3.4
14	Reporting bias assessment	Describe any methods used to assess risk of bias due to missing results in a synthesis. Section 2.6; funnel-plot testing was not performed and the reason is stated.	Section 2.6
15	Certainty assessment	Describe any methods used to assess certainty in the body of evidence for an outcome. Section 2.6.	Section 2.6; Supplementary Methods
	RESULTS		
16a	Study selection	Describe the results of the search and selection process, from the number of records identified to the number of studies included, ideally using a flow diagram. Section 3.1 and Figure 1.	Section 3.1; Figure 1; Supplementary Table 2
16b	Excluded studies	Reports excluded from frequency pooling are discussed with reasons in Sections 2.5 and 3.2. Supplementary Table 2 reconciles original eligibility decisions; Supplementary Data 2 provides the expanded record-level decision audit.	Sections 2.5, 3.2; Supplementary Tables 2, 5
17	Study characteristics	Cite each included study and present its characteristics. Section 3.2 and Table 1.	Section 3.2; Tables 1–2
18	Risk of bias in studies	Present assessments of risk of bias for each included study. Section 3.6 and Figure 4; item wording in Supplementary Table 4.	Section 3.6; Figure 4; Supplementary Tables 3–4
19	Results of individual studies	For all outcomes, present for each study summary statistics for each group and an effect estimate with its precision. Table 1 and Figure 2.	Table 1; Figure 2
20a	Results of syntheses	For each synthesis, briefly summarise the characteristics and risk of bias among contributing studies. Sections 3.2 and 3.6.	Sections 3.2, 3.6
20b	Results of syntheses	Present results of all statistical syntheses conducted, including summary estimates and their precision and heterogeneity measures. Section 3.3 and Figure 2.	Sections 3.3–3.4; Figure 2
20c	Results of syntheses	Present results of all investigations of possible causes of heterogeneity among study results. Section 3.4.	Sections 3.4, 3.6
20d	Results of syntheses	Present results of all sensitivity analyses conducted to assess robustness of the synthesised results. Sections 3.4 and 3.6.	Sections 3.4, 3.6
21	Reporting biases	Present assessments of risk of bias due to missing results for each synthesis assessed. Sections 3.6 and 4 (case-report publication bias).	Sections 3.6, 4
22	Certainty of evidence	Present assessments of certainty in the body of evidence for each outcome assessed. Section 2.6 and Section 4.	Sections 2.6, 4; Supplementary Methods
	DISCUSSION		
23a	Discussion	Provide a general interpretation of the results in the context of other evidence. Section 4.	Section 4
23b	Discussion	Discuss any limitations of the evidence included in the review. Section 4 (Limitations).	Section 4.3

Item #	Section / topic	Checklist item and where it is addressed	Location
23c	Discussion	Discuss any limitations of the review processes used. Section 4 (Limitations).	Sections 2.3, 4.3
23d	Discussion	Discuss implications of the results for practice, policy and future research. Sections 4 and 5.	Sections 4, 5
	OTHER INFORMATION		
24a	Registration and protocol	Provide registration information for the review, including the register name and registration number. PROSPERO CRD420261458659.	Abstract; Section 2.1
24b	Registration and protocol	Indicate where the review protocol can be accessed, or state that a protocol was not prepared.	Section 2.1
24c	Registration and protocol	Describe and explain amendments to registered information. The Gurram eligibility exception and post hoc analyses are explicitly disclosed.	Sections 2.1, 2.2, 2.5, 2.7
25	Support	Describe sources of financial or non-financial support and the role of the funders. Declarations, Funding.	Funding
26	Competing interests	Declare any competing interests of review authors. Declarations, Conflict of Interest.	Conflict of Interest
27	Availability of data, code and other materials	Report which of the following are publicly available and where: template data collection forms; data extracted; data used in all analyses; analytical code; any other materials. Declarations, Data Availability Statement; analysis code and study-level counts provided as Supplementary Data 1; search strategies, exclusion table and risk-of-bias appraisal in Supplementary Material 1; screening log, reviewer-agreement data and reconciled extraction dataset available from the corresponding author on request.	Data Availability Statement; Supplementary Material; Supplementary Data 1

Reference: Page MJ, McKenzie JE, Bossuyt PM, Boutron I, Hoffmann TC, Mulrow CD, et al. The PRISMA 2020 statement: an updated guideline for reporting systematic reviews. BMJ (2021) 372:n71. doi: 10.1136/bmj.n71
